# Elective Submandibular Gland Resection in Patients with Squamous Cell Carcinomas of the Tongue

**DOI:** 10.22038/ijorl.2020.44283.2463

**Published:** 2021-01

**Authors:** Shadi Javadi, Bijan Khademi, Mohammad Mohamadianpanah, Mahmoud Shishegar, Amirhossein Babaei

**Affiliations:** 1 *Otolaryngology Research Center, Shiraz University of Medical Sciences, Shiraz, Iran.*; 2 *Department of Otolaryngology, Khalili Hospital, Shiraz University of Medical Sciences, Shiraz, Iran.*; 3 *Colorectal Research Center, Shiraz University of Medical Sciences, Shiraz, Iran.*; 4 *Student Research Committee, Shiraz University of Medical Sciences, Shiraz, Iran.*

**Keywords:** Glossectomy, Lymph Node, Mouth, Submandibular Gland, Squamous Cell Carcinoma

## Abstract

**Introduction:**

Submandibular gland resection is a controversial issue in patients with oral tongue squamous cell carcinomas (SCC). This study aimed to determine the frequency of submandibular gland involvement in patients who had undergone elective submandibular gland resection following oral tongue SCC.

**Materials and Methods:**

This cross-sectional retrospective study was performed between 2001 and 2017 on patients with oral tongue SCC who had undergone glossectomy in a referral center for otorhinolaryngology surgery, Shiraz, Iran.

**Results:**

In this study, 131 patients were included. Their mean age was 59.84 years (range: 19-86). The mean tumor size was 2.83 cm (range 0.3-7). The vast majority (92%) of the patients were at stage III-IVa and had well (55%) to moderate (31%) differentiated tumor. The mean diameter of the submandibular gland was 3.87 cm (range: 1.5-6 cm). There was only one (0.76%) patient with submandibular involvement. She was an 80-year-old woman with a T2 well differentiated tumor without cervical lymph node involvement in the neck node dissection.

**Conclusion:**

In patients with oral tongue SCC, submandibular gland involvement is rare and its elective resection in not recommended.

## Introduction

Oral tongue squamous cell carcinoma (SCC) has unique clinical behavior compared to other head and neck sites. Oral tongue patients with a more than 20% rate of occult cervical metastasis will benefit from either surgical or radiotherapeutic management of the neck (1). Submandibular gland involvement is an important issue in treatment of SCC. Several studies showed that the rate of metastasis to submandibular gland is very low even in the presence of level IB lymph nodes involvement (2). The treatment option for many of the patients with oral tongue SCC is resection of the primary tumor, and then selective neck dissection of levels I to III/IV. The submandibular gland is typically resected due to the following reasons: probable submandibular gland involvement; removal of the involved level IB lymph nodes; accelerated dissection of level IB; and complete dissection of level IB lymph nodes (3). Forasmuch as submandibular gland is the main secretor of saliva in the oral cavity, xerostomia is the most important side effect of resection of submandibular glands (4). The prophylactic surgical resection of the submandibular gland is controversial (5). Nowadays, some studies have stated that the prophylactic resection of submandibular glands is not necessary in patients with oral tongue SCC (2,6). The confirmation of this fact can help the surgeons to prevent unnecessary excision of the submandibular gland. In addition to side effects like xerostomia, resection of submandibular gland is difficult due to its adjacent structures such as marginal mandibular, hypoglossal and lingual nerves. This study aimed to investigate the frequency of submandibular gland involvement in patients with oral tongue SCC who had undergone elective submandibular gland resection.

## Materials and Methods

This cross-sectional retrospective study was performed on patients with oral tongue SCC who had undergone glossectomy in Khalili hospital, a referral center for otorhinolaryngology surgery, Shiraz University of Medical Sciences (SUMS), Shiraz, Iran between 2001 and 2017. All patients who were referred to Khalili hospital with histopathologically confirmed oral tongue SCC were enrolled in this study. Glossectomy was performed using cutting diathermy and tumor-free margin achieved. Patients who were not followed up, had not been operated, or did not agree to participate in this study were excluded from the study. Also, patients with no microscopically confirmed pathology and incomplete medical records were excluded from the study.Patient’s information including age, sex, histopathology of SCC, size of tumor, size and level of the lymph node, microscopic invasion of the lymph node, size of the submandibular gland, tumoral involvement of the submandibular gland, TNM (tumor, nodule, metastasis) staging, and 2-year local recurrence was gathered from their medical records in the archive of the hospital. The largest recorded dimension of the dissected tissue (tumor, lymph node, submandibular gland) was used as the tissue size for statistical analysis. Before the study, the protocol and the patient informed consent form were reviewed and approved by the local Ethics Committee of SUMS (IR.sums.med.rec.1396.s39). The study was conducted according to the principles of the Declaration of Helsinki (1996) and Good Clinical Practice Guidelines (1996). All subjects signed a general written informed consent form before operation to permit using the data of their medical records while considering their privacy. Normal distribution of data was assessed by Shapiro-Wilk test. Descriptive statistics for categorical and continuous variables were reported as frequency (percent) and mean (SD), respectively. Independent t-test or Mann-Whitney U Test were used for comparison of continuous variables. The relationship between the two categorical variables was determined using the Chi-square test or the Fisher's exact test. The analysis was conducted using SPSS 25 software (SPSS Inc., Chicago, IL, USA). *P*-values less than 0.05 were considered to be statistically significant.

## Results

In this study, 131 patients were included. The mean age of the patients was 59.84 years (range: 19-86). Mean tumor size was 2.83 cm (range 0.3-7 cm). Mean diameter of submandibular gland was 3.87 cm (range 1.5-6 cm). The mean, range and standard deviation of age, diameter of tumor, diameter of lymph node, and diameter of submandibular gland are shown in Table 1.

**Table 1:** The participants’ baseline data

**Variable**	**Mean**	**Minimum**	**Maximum**	**Standard deviation**
Age (year)	59.84	19	86	16.61
Diameter of tumor (cm)	2.83	0.3	7	1.53
Diameter of lymph node (cm)	2.07	0.4	8.5	1.07
Diameter of submandibular gland (at the largest diameter) (cm)	3.87	1.5	6	0.65

Patient’s demographic data and data about tumor characteristics are shown in Table 2.

**Table 2:** Patient’s demographic data and tumor characteristics

**Variable**	**Condition**	**Frequency**	**Percent**
Gender	Male	63	48.09%
	Female	68	51.91%
Tumor side	Ipsilateral (right)	54	41.22%
	Ipsilateral (left)	75	57.25%
	Bilateral	1	0.76%
	Missing	1	0.76%
Histopathology	Well differentiated SCC	72	54.96%
	Moderately differentiated SCC	41	31.30%
	Poorly differentiated SCC	7	5.34%
	Microinvasive differentiated SCC	7	5.34%
	SCC (no type was mentioned)	3	2.29%
	Verrucous carcinoma	1	0.76%
Lymph node dissection	Level I to III	49	37.40%
	Level I to IV	60	45.80%
	Level I to V	13	9.92%
	Bilateral neck dissection	4	3.05%
	I to III bilateral neck dissection	1	0.76%
	Radical neck dissection	3	2.29%
	Not performed	1	0.76%
Lymph node involvement	Yes	54	41.22%
	No	76	58.02%
	Missing	1	0.76%
TNM staging	III	105	80.15%
	IVa	16	12.21%
	IVb	2	1.53%
	Missing (lymph node size was not recorded)	8	6.11%
Local recurrence	Yes	32	24.43%
	No	99	75.57%

SCC: Squamous Cell Carcinomas.

Table 3 shows TNM staging of the participants. The vast majority (92%) of the patients were at stage III-IVa. 

Fifty-five percent of all SSC were well differentiated and 31% were moderately differentiated.

**Table 3:** The patients’ TNM staging

**TNM**	**Stage**	**Frequency**	**Percent**
T1N1M0	III	25	19.08%
T1N2M0	IVa	6	4.58%
T2N1M0	III	48	36.64%
T2N2M0	IVa	5	3.82%
T2N3M0	IVb	1	0.76%
T3N1M0	III	32	24.43%
T3N2M0	IVa	5	3.82%
T3N3M0	IVb	1	0.76%
No LN size	-	8	6.11%

The relationship of the recurrence and lymph node, involvement, size of tumor and size of submandibular gland is presented in Table 4. As shown, there was no statistically significant relationship between the recurrence and size of tumor, lymph node, and submandibular gland.

**Table 4:** Relationship between recurrence and other factors

**Variable**		**Recurrence**		***p*** **-value**
		**Yes**	**No**	
Lymph node involvement	Yes	16	38	0.645*
	No	20	57	
Size of tumor (cm)		2.809	2.842	0.980**
Size of lymph node (cm)		2.050	2.078	0.640**
Size of submandibular gland (cm)		3.972	3.830	0.090**

*Chi square ** Mann-Whitney U test

There was a statistically significant relationship between the lymph node involvement and size of the tumor (P=0.025) and size of the lymph node (P= 0.018). Table 5 shows the relationship between the lymph node involvement and size of tumor, lymph node, and submandibular gland.

**Table 5:** The relationship between the lymph node involvement and other factors

**Variable**	**Lymph node involvement**		***p*** **-value**
	**Yes**	**No**	
Size of tumor (cm)	3.208	2.569	0.025*
Size of lymph node (cm)	2.370	1.839	0.018*
Size of submandibular gland (cm)	3.759	3.949	0.090*

* Mann-Whitney U test

There was only one (0.76%) patient with submandibular gland involvement. She was an 80-year-old woman with a T2 well differentiated tumor (4×2.5×1.5 cm left sided) without cervical lymph node involvement in the neck dissection. Maximum diameter of the lymph node was 1.5 cm. The size of the submandibular gland was 3×2.5×1 cm. No recurrence was observed in this patient.

Because of the low prevalence, no relationship could be identified between submandibular gland involvement and age, sex, lymph node involvement, zone of lymph node involvement, size of lymph nodes, recurrence of disease, and TNM stage of the disease.

## Discussion

In this study, we determined the submandibular gland involvement in patients with oral tongue SCC. There was only one (0.76%) patient with submandibular gland involvement. Also, the rate of lymph node involvement was 41.22%.

The removal of even one submandibular gland led to diminished salivary flow and subsequently xerostomia. Also, patients were susceptible to dental caries and increased risk of oral candidiasis (7). Moreover, the undesirable influence on talking, swallowing, and tasting can lead to a reduced quality of life (8). The low rate of submandibular gland involvement can be due to its fibrous capsule and relative absence of lymphatic and vascular drainage (9). 

Another important issue about submandibular gland is the effect of adjuvant radiotherapy on the salivary flow. Although some studies have not revealed any relationship between dissection of the submandibular glands and xerostomia (10), some others suggest gland-sparing radiotherapy modalities for patients who have undergone adjuvant radiotherapy after surgical treatment (11,12).However, patients with preserved submandibular gland showed better salivary flow even with adjuvant radiotherapy (3). In our study, there was no statistically significant relationship among the recurrence and size of tumor, lymph node, and submandibular gland. These findings were in contrast with those of previous studies. Several studies showed that increased tumor size was associated with increased rate of recurrence (13-15). The rate of submandibular gland involvement in our study was 0.76%. Previously published articles reported a rate of less than 4.6% (3). A study by Malgonde and Kumar reported that among the 98 dissected submandibular glands only 3 patients (3.06 %) had involvement of submandibular gland. They suggest that decision about dissection of the gland should be made during the surgery by means of frozen sections and gross examination (16).

Agarwal et al. conducted a study to evaluate the metastasis to the submandibular gland in individuals with oral cavity SCC. From all 112 patients included in the study, no submandibular gland involvement was reported in any of the cases. They concluded that preservation of submandibular glands could be a good technique for decreasing the side effect of neck dissection (9).

Another study by Panda et al. found that 6 out of 163 removed submandibular glands (3.68%) showed involvement. Four of the six involved glands showed direct contiguous spread from the primary tumor, one showed extra-capsular spread from the level IB lymph nodes, and another one had evidence of both modes of spread. No glands had evidence in favor of metastasis (0%). They recommended that submandibular gland could be safely preserved during neck dissections for oral SCC. The only exception of this recommendation is high suspicion to its involvement such as juxtaposition of the primary tumor to the gland, radiographic evidence of intra-capsular lymph node, gross signs of invasion to the submandibular gland, and salvage surgeries in patients with previous history of radiotherapy or recurrence (17).

Malik et al. investigated the submandibular gland involvement in patients with oral cavity carcinoma among 137 patients. They found that metastasis in N0 level deep to the submandibular gland was seen in only one case with clinically node negative neck. None of the patients had a primary tumor of the tongue.

They concluded that, even in presence of level IB metastasis, excision of level IB can be done without removal of the submandibular gland. Moreover, in early tongue tumors, dissection of the submandibular gland at level IB was not obligatory as no metastases were seen deep to the submandibular glands (18). An investigation by Cakir et al. retrospectively reviewed 183 neck specimens. Direct invasion to the submandibular gland was seen in two specimens. The only risk factor for submandibular gland involvement was tumors in the floor of the mouth, which were more susceptible to submandibular gland invasion. These results recommend that submandibular gland could be preserved safely in early stages of oral cavity carcinoma, except for the tumor in the floor of the mouth (19).

A systematic review by Dundar et al. reviewed 17 articles to assess the rate of submandibular gland involvement by oral cavity and oropharynx SCC. They analyzed 2792 submandibular gland resections from 2306 patients. Of these, 58 specimens (2.0%) showed tumor involvement. 

They claimed that preservation of submandibular gland could be possible in these patients according to infrequent submandibular gland involvement. Nevertheless, further studies are recommended to determine the function of preserved submandibular gland among patients who have undergone adjuvant radiotherapy (3).

The advantage of our research was the long period of study. Also, the rate of recurrence was documented and reported in our study. However, our study had some limitations. Although we continued our study for a long period of time, because of low prevalence of oral tongue SCC, the number of patients with submandibular gland involvement was very low. Accordingly, we suggest that the researchers should perform a meta-analysis on previously published articles to determine the conditions in which resection of submandibular gland is necessary. This helps the surgeons to select patients who need submandibular gland dissection and prevent unnecessary resection.

## Conclusion

In patients with oral tongue SCC, submandibular gland involvement is rare. We suggest that it is not necessary to resect submandibular gland routinely in patients who had undergone neck dissection due to oral tongue SCC.
